# Cardiac steatosis and left ventricular remodeling in heart failure with reduced and preserved ejection fraction

**DOI:** 10.1186/1532-429X-17-S1-P309

**Published:** 2015-02-03

**Authors:** Masliza Mahmod, Nikhil Pal, Cameron Holloway, Vanessa M Ferreira, Sairia Dass, Jane M Francis, Oliver Rider, Theodoros D Karamitsos, Houman Ashrafian, Stefan Neubauer

**Affiliations:** 1 Division of Cardivascular Medicine, Radcliffe Department of Medicine, Oxford Centre for Clinical Magnetic Resonance Research, Oxford, UK; 2 1st Department of Cardiology, AHEPA Hospital, Aristotle University of Thessaloniki, Thessaloniki, 54636, Greece

## Background

Heart failure (HF) is characterised by alterations in fatty acid and glucose metabolism. We aimed to determine if myocardial lipid is increased in HF with reduced (HFrEF) and preserved (HFpEF) ejection fraction (EF), and assess whether it is related to cardiac structure and function.

## Methods

25 HFrEF due to dilated cardiomyopathy (DCM), 18 HFpEF (defined by EF >50%, abnormal diastolic function, maximum oxygen consumption <80% predicted for age, height and gender, with a cardiac limitation in exercise) and 28 normal volunteers were prospectively recruited. All subjects underwent cardiovascular magnetic (MR) resonance at 3T for the determination of left ventricular (LV) volumes and function, and cardiac ^1^H MR spectroscopy to quantify myocardial lipid/water (%).

## Results

**Figure 1:**
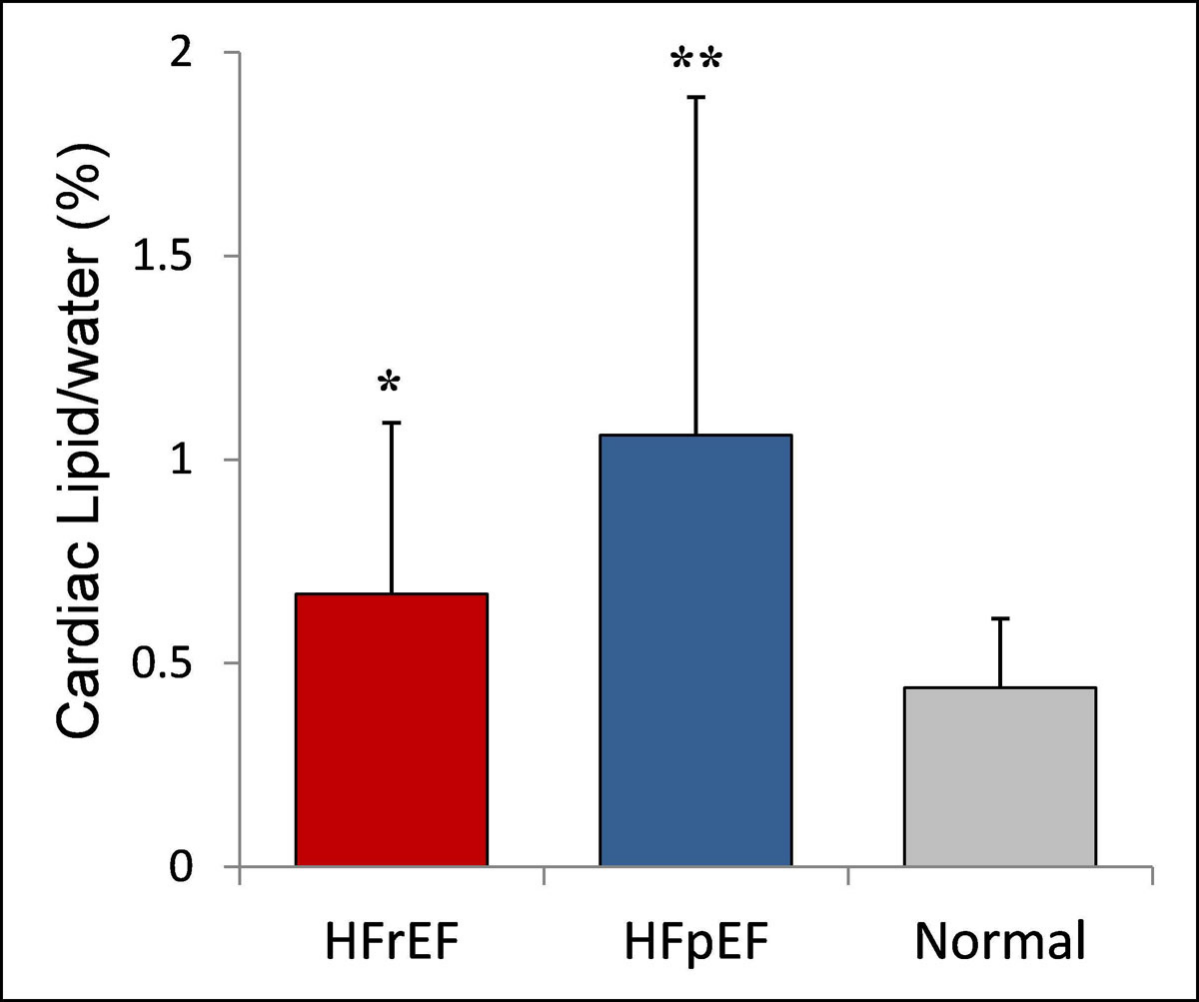
**Cardiac lipid content in HFrEF, HFpEF and normal subjects.** *p<0.05 vs HFpEF and normal; **p<0.05 vs normal.

As expected DCM patients had significantly increased LV volumes and reduced EF, whilst HFpEF patients had significantly increased LV mass to end-diastolic volume ratio (LV mass/EDV). Importantly, cardiac lipid was increased in both HFrEF and HFpEF when compared to normal controls (cardiac lipid/water 0.67±0.42% in HFrEF; 1.06±0.83% in HFpEF versus normal controls 0.44±0.17, all p<0.05), with HFpEF group having the highest level of cardiac lipid (Table 1, Figure 1). In DCM patients, cardiac lipid negatively correlated with LVEF (r=-0.33, p=0.03) and positively correlated with LV size (r=0.54, p<0.001). In HFpEF, cardiac lipid positively correlated with age (r=0.41, p=0.008) and LV mass/EDV (r=0.37, p=0.02). Although HFpEF patients were significantly older, with age positively correlated with cardiac lipid, multiple regression analysis showed that age is not an independent predictor of cardiac lipid.

**Table 1:** Clinical characteristics of patients with reduced (HFrEF), preserved (HFpEF) ejection fraction and normal controls.

	HFrEF (n = 25)	HFpEF (n = 18)	Normals (n = 28)	P value
Age (years)	60 ± 11*	74 ± 6**	61 ± 5	<0.001

Female, n (%)	8 (32)	12 (67)	12 (43)	0.075

NYHA class, n (%)				

I	3 (12)	0	0	-

II	21 (84)	18 (100)	0	-

III	1 (4)	0	0	-

Body mass index (kg/m2)	28 ± 5	28 ± 6	27± 4	0.29

E'	-	4.5 ± 1.2	13.1 ± 3.5	0.001

E/E' ratio	-	10.7 ± 2.7	7.6 ± 3.0	0.002

E/A ratio	-	0.7 ± 0.26	1.0 ± 0.3	0.001

Blood glucose	5.4 ± 1.0	5.5 ± 1.1	5.2 ± 1.0	0.55

Free fatty acids	0.44 ± 0.22	0.49 ± 0.29	0.54 ± 0.30	0.46

Triglycerides (mmol/L)	1.5 ± 0.7	1.2 ± 0.4	1.1 ± 0.3	0.12

Low-density lipoprotein (mmol/L)	2.9 ± 0.9	2.4 ± 0.7	3.1 ± 1.1	0.07

High-density lipoprotein (mmol/L)	1.4 ± 0.5	1.6 ± 0.4	1.4 ± 0.4	0.49

CMR findings				

Cardiac lipid/water (%)	0.67 ± 0.42†	1.06 ± 0.83**	0.44 ± 0.17	0.001

LV end-diastolic volume (ml)	233 ± 82†	111 ± 21‡	144 ± 28	<0.001

LV ejection fraction (%)	38 ± 9†	74 ± 6‡	69 ± 5	<0.001

LV mass index (g/m2)	80 ± 25†	54 ± 12‡	55 ± 11	<0.001

LV mass/EDV (g/mL)	0.69 ± 0.18*	0.91 ± 0.22**	0.74 ± 0.16	0.003

* p<0.05 vs HFpEF and p>0.05 vs normal; ** p<0.05 vs normal.

† p<0.05 vs HFpEF and normal; χ‡ p>0.05 vs normal.

## Conclusions

This is the first study to demonstrate that myocardial steatosis occurs in both HFrEF and HFpEF and is related to parameters of LV remodeling. This suggests that myocardial lipid may play a role in the pathophysiological processes of LV remodeling in both HFrEF and HFpEF. Cardiac lipid accumulation may be a potential therapeutic target in these conditions.

## Funding

N/A.

